# Juvenile food limitation in standardized tests: a warning to ecotoxicologists

**DOI:** 10.1007/s10646-012-0973-5

**Published:** 2012-07-28

**Authors:** Elke I. Zimmer, T. Jager, V. Ducrot, L. Lagadic, S. A. L. M. Kooijman

**Affiliations:** 1 Department of Theoretical Biology, Faculty of Earth & Life Sciences, Vrije Universiteit, de Boelelaan 1085, 1081 HV Amsterdam, The Netherlands; 2 INRA, Equipe Ecotoxicologie et Qualité des Milieux Aquatiques, UMR0985 Ecologie et Santé des Ecosystèmes, Agrocampus Ouest, 65 rue de Saint Brieuc, 35042 Rennes, France

**Keywords:** *Lymnaea stagnalis*, Dynamic Energy Budget, Toxicity, Growth curve, Multiple stress, Nutritional status

## Abstract

Standard ecotoxicological tests are as simple as possible and food sources are mainly chosen for practical reasons. Since some organisms change their food preferences during the life-cycle, they might be food limited at some stage if we do not account for such a switch. As organisms tend to respond more sensitively to toxicant exposure under food limitation, the interpretation of test results may then be biased. Using a reformulation of the von Bertalanffy model to analyze growth data of the pond snail *Lymnaea stagnalis*, we detected food limitation in the early juvenile phase. The snails were held under conditions proposed for a standardized test protocol, which prescribes lettuce as food source. Additional experiments showed that juveniles grow considerably faster when fed with fish flakes. The model is based on Dynamic Energy Budget (DEB) theory, which allows for mechanistic interpretation of toxic effects in terms of changes in energy allocation. In a simulation study with the DEB model, we compared the effects of three hypothetical toxicants in different feeding situations. The initial food limitation when fed with lettuce always intensified the effect of the toxicants. When fed with fish flakes, the predicted effect of the toxicants was less pronounced. From this study, we conclude that (i) the proposed test conditions for *L. stagnalis* are not optimal, and require further investigation, (ii) fish flakes are a better food source for juvenile pond snails than lettuce, (iii) analyzing data with a mechanistic modeling approach such as DEB allows identifying deviations from constant conditions, (iv) being unaware of food limitation in the laboratory can lead to an overestimation of toxicity in ecotoxicological tests.

**Electronic supplementary material:**

The online version of this article (doi:10.1007/s10646-012-0973-5) contains supplementary material, which is available to authorized users.

## Introduction

Selecting a single food type for test organisms throughout the test duration can be a challenge, especially in full life-cycle tests. Clearly, we do not try to reproduce the natural environment in the laboratory. The test conditions for the species of interest (light regime, food availability, temperature, etc.) are standardized to conditions which are easy to replicate and have proven to maintain the species in good condition. These conditions are kept as constant as possible to facilitate the interpretation of test results: only under constant conditions, we can distinguish the effects caused by the chemical of interest from any side effects resulting from the experimental conditions. Since we usually do not know exactly what the test organisms eat in nature, food is mainly chosen for practical reasons: e.g., the pond snail *Lymnaea stagnalis* is usually fed with lettuce (Ducrot et al. 2010b). However, we know that a range of species change their dietary preferences during the life cycle: organisms mainly require proteins to build up new structures, and during the rapid growth in the juvenile stage, the protein requirements are higher than in the adult stage. Well known examples are mammals, that feed their young on protein-rich milk after birth, and chicken and ducks, which feed on insects directly after hatching, but switch to a mainly vegetarian diet as adults. For some ecotoxicological standard test protocols, the switch in diet has been accounted for already, e.g. in the early life-stage toxicity test for fish (OECD 1992). For each of the five recommended species, detailed information is provided regarding food item(s) for the newly hatched fish, the juveniles, and the adults.

If we aim to maintain the test organisms at constant ad libitum feeding conditions, we aim to do so during the whole experiment. A deviation from the ad libitum feeding situation results in stress by food limitation. Heugens et al. (2001) reviewed how additional stressors such as nutritional state influence the toxicity of test compounds to aquatic organisms. A decrease in food level usually increases toxicity: depending on the nutritional state of the organism, the toxicity can be 10-fold higher in comparison to well-fed organisms (Heugens et al. 2001).

If the nutritional status is so important for the sensitivity of organisms, how can we monitor it? One simple way to test for the constancy of environmental conditions for organisms, such as the availability of proper food, is to scrutinize the growth curves. It was observed nearly a century ago by Ludwig von Bertalanffy (1934) that the change in length over time for most organisms follows a certain shape under constant conditions: when expressed as a length measure, growth is continuous, linear in the beginning, and approaches a maximum size asymptotically. The von Bertalanffy pattern applies for most animals under constant conditions (Kooijman 2010). Therefore, a deviation of the growth curve from this pattern points to a deviation from constant conditions for the organism in its environment. For example, for bacterivorous nematodes, it was found that the deviating growth curves could be explained by juvenile food limitation: the mouth parts of newly hatched worms do not allow efficient feeding on the bacterial food provided in experimental tests (see Jager et al. 2005).

Investigations on the toxicity of chemicals generally aim to assess the impact caused by the chemicals in the environment, where organisms hardly ever experience constant conditions. We therefore need mechanistic approaches that take into account interactions between toxicants and environmental factors such as food conditions and/or temperature. Mechanistic modeling approaches receive more and more attention in this context (Grimm et al. 2009; Preuss et al. 2009).

In this study, we combined experiments and modeling to investigate juvenile food limitation, and assess the potential for bias in the interpretation of ecotoxicological test results. We chose the great pond snail (*L. stagnalis*) as model organism, because it has been identified as a relevant candidate species for the development of toxicity test guidelines by the OECD (2010). The proposed laboratory conditions are currently under investigation regarding their suitability for standardization. While investigating growth data from a full life-cycle experiment (Ducrot et al. 2010a), we found a deviation from the von Bertalanffy pattern. We hypothesize that this deviation is due to food limitation in the early juvenile phase. We use a reformulation of the von Bertalanffy growth model that is applied in Dynamic Energy Budget (DEB) theory (e.g., Kooijman et al. 2008), because it allows for mechanistic interpretation of toxic effects and their interaction with food availability. In DEB, the effect of a toxicant can be understood as a change in the acquisition or allocation of resources (Jager and Zimmer 2012). We used existing partial and full life-cycle data on different food levels without toxicant exposure to parameterize an individual model for the pond snail. To test our hypothesis of juvenile food limitation, we conducted experiments with different types and amounts of food with freshly hatched snails. Using model simulations with hypothetical toxicants, we investigated the combined effect of juvenile food limitation and toxicity on the interpretation of ecotoxicological experiments.

## Materials and methods

### Growth model

The von Bertalanffy growth model has been shown to apply to the pond snail *L. stagnalis* by Zonneveld and Kooijman (1989). The shell length *L* (in cm) is given as

1 $$ \frac{dL}{dt}= \dot{r}_B (L_{\infty} - L), $$

where $\dot{r}_B$ is the von Bertalanffy growth rate constant (in $\hbox{d}^{-1}$), and *L*_∞_ is the maximum shell length at abundant food. The growth rate constant $\dot{r}_B$ determines how fast the organism reaches its maximum size *L*_∞_. The parameters of Eq. 1 are only constant for organisms that experience constant conditions. A change in food level will both affect $\dot{r}_B$ and *L*_∞_. To explain these effects, we use the reformulation of the von Bertalanffy growth model that is used in DEB theory (Kooijman et al. 2008).

DEB theory provides a conceptual framework that explains how organisms allocate energy from food into growth, reproduction, development, and maintenance. The same framework can be applied to all organisms; inter-species differences are mainly expressed as differences in parameter values. The model parameters determine how much energy is invested in which process, i.e. how expensive one process is relative to the others. We will here only shortly mention the parameters that are important for the present work, i.e. the parameters that play a role in the growth model. A more detailed description of the model and the underlying assumptions can be found in the Online Resource, Sects. 1.1 and 1.2. For more introduction on DEB theory, we refer the reader to Van der Meer (2006), and for a deeper insight, to Kooijman (2010). The two parameters of the von Bertalanffy equation can be expressed in terms of DEB parameters as

2 $$ L_m = \frac{\dot{v}}{\dot{k}_{M} g}, \quad L_{\infty} = f L_m \quad \hbox{and} \quad \dot{r}_B = \frac{g \dot{k}_M}{3(f + g)}, $$

which reveals how *L*_∞_ and $\dot{r}_B$ are linked to each other and to the food level. In Eq. 2, the following DEB parameters appear: *g*, the energy investment ratio (−), $\dot{v}$, the energy conductance $(\hbox{cm d}^{-1}), \dot{k}_M$, the somatic maintenance rate coefficient $(\hbox{d}^{-1})$, and the scaled functional response *f* (−). The scaled functional response *f* is the actual ingestion rate of an animal divided by the maximum ingestion rate for its size. For an individual under ad libitum feeding conditions, *f* = 1, whereas for a starving individual, *f* = 0, so that for limiting conditions 0 < *f* < 1. Note that we assume fast reserve dynamics and thus use the scaled functional response *f* in $\dot{r}_B$ instead of the scaled reserve density *e* (see Online Resource Sect. 1.2, Fig. S2). How these parameters are linked to metabolic processes is explained in detail in Jager and Zimmer (2012).

Metabolic rates are known to depend on temperature, and usually, the Arrhenius relation provides a good description for the temperature dependence across species (Gillooly et al. 2006). In DEB, this is accounted for: all parameters that have the dimension per time $(\hbox{d}^{-1})$ are multiplied by a temperature correction factor that can be derived from the Arrhenius relation (see Freitas et al. (2007) for the procedure). We need to account for the temperature dependence of the parameters because one of the experiments (see description below) has been conducted at a different temperature than the others. For the Arrhenius temperature, we use a typical value of 11900 K (see Kooijman 2010, and Online Resource, Sect. 1.4).

**Fig. 1:**
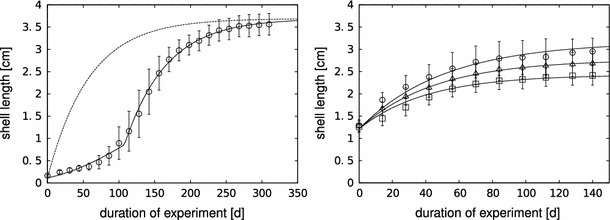
The growth curves obtained in the full life-cycle experiment (FLE,* left panel*) and the partial life-cycle experiment (PLE,* right panel*). The* symbols* are the mean values of the measured shell length, the* error bars* the corresponding standard deviations. The* lines* correspond to the model predictions: the* dashed line* is the prediction without the juvenile food limitation function for the FLE.* Right panel*: $\circ$ ad libitum lettuce, $\triangle$ 50 % of ad libitum, $\square$ 25 % of ad libitum

To describe the effect of juvenile food limitation on growth, we make the scaled functional response *f* a function of body size. For simplicity, we use a linear function for *f* related to length:

3 $$ f(L) = a f_{0} \frac{L}{L_{m}} \qquad \hbox{for} \qquad L < L_f, $$

where *a* is the *food quality factor*. This is a dimensionless constant, which relates to the quality of the provided food source. Note that we use the normalized length (*L* / *L*_*m*_) to keep *a* dimensionless. The parameter *f*_0_ is the scaled functional response for *L* > *L*_*f*_ in that treatment. It will be set to one for ad libitum feeding with lettuce, and for the highest amount of fish flakes given in the juvenile feeding experiment (see description below). The growth of the snails follows the von Bertalanffy pattern after they reach a certain shell length (see Fig. 1). We call this length the critical length $L_f(\hbox{cm})$, and assume that above that size, the snails are not limited by food quality anymore (i.e. *f* = *f*_0_ in the model).

### Simulation experiments with hypothetical toxicants

The DEB model allows for mechanistic interpretation of toxic effects, which enter the model as changes in parameter values (Jager et al. 2010). To study the interaction between toxicants and juvenile food limitation, we use model simulations where we include different metabolic mechanisms of action (mMoA) in terms of DEB. The inclusion of toxic effects in the DEB model is explained in detail in Jager and Zimmer (2012). Each mMoA leads to a specific combination of effect patterns (e.g. effects on growth, reproduction, development, feeding, respiration). All scenarios were run with the control value for all model parameters (i.e., the parameters that were estimated from experiments without toxicants), and with a range of values to simulate stress due to three hypothetical toxicants with different mMoAs. We use the three general mMoAs that affect growth in the context of DEB theory: (1) an increase of costs for somatic maintenance (i.e., $\uparrow \dot{k}_M$), (2) a decrease of assimilation efficiency (i.e., ↓ *f*), and (3) an increase of costs for growth (i.e., $\downarrow \dot{k}_M, \uparrow g$) (see Jager and Zimmer (2012), and the Online Resource, Sect 1.3, and Table 2). We assumed that a given toxicant would only impact one metabolic process. For the effect on assimilation and the effect on maintenance, the range of increase was simulated up to 15 %, while for the effect on costs for growth, it was simulated up to 600 %. These mMoAs have been found in several analyses of ecotoxicological data sets, for example in the nematodes *Acrobeloides nanus* and *Caenorhabditis elegans*, which were tested with carbendazim, cadmium, pentachlorobenzene, and aldicarb (Alda Álvarez et al. 2006; Wren et al. 2011). For example, in the highest exposure concentration with cadmium, the effect on the growth costs on *A. nanus* was predicted to increase from some 200 % up to 600 % over the life cycle. We additionally implemented three feeding scenarios: (A) individuals under true ad libitum feeding (*f*(*L*) = 1 throughout the life-cycle), (B) individuals with food limitation in the juvenile phase (0 < *f*(*L*) < 1), which were assumed to be fed with lettuce and (C) individuals with food limitation in the juvenile phase, which were assumed to be fed with fish flakes. In combination with the three different mMoAs, we therefore have nine different simulation studies. To facilitate the comparison with the life-cycle experiments, the duration of the simulations was 400 days.

### Description of available data: the full life-cycle (FLE) and the partial life-cycle (PLE) experiments

To determine the DEB parameters that govern growth, it is essential to have data on body size over the life-cycle at different food levels (Kooijman et al. 2008). We used data from a full life-cycle experiment (336 days, from now on FLE) at ad libitum food, and data from a partial life-cycle experiment (184 days, from now on PLE) at three different food levels for the parameterization. Those experiments had been conducted earlier for other projects at the French National Institute for Agricultural Research, INRA, in Rennes. The experimental protocol, the setup, and part of the data of the FLE have been published by Ducrot et al. (2010a). The PLE has been conducted using a very similar protocol and setup, where only the initial conditions (i.e., initial age and size of snails) and the feeding conditions were slightly different. Therefore, we only give a short description of both experiments here. All snails mentioned in the present work originate from the culture at INRA in Rennes. A detailed description of the culture conditions and the experiments as well as the data used for the parameterization of the model can be found in the Online Resource, Sect. 2.

The FLE was conducted to assess the effects of diquat on the life-cycle of *L. stagnalis* (Ducrot et al. 2010a). We used the growth data of the controls in the present work. The whole experiment was conducted under a photoperiod of 14/10 L/D at 21 ± 1 °C. Freshly hatched snails were transferred to the test vessels in groups of five (24 replicates). They were fed abundantly with weighted slices of organic lettuce (*Lactuca sativa*), but only if no leftover remained, to avoid spoiling the water quality due to the degradation of leftovers. The freshly hatched snails were fed with one slice of 21 mmØ. The number of slices of lettuce given was doubled when half of the replicates had fully consumed the previously provided lettuce on the next day. The test vessel volume was gradually increased along the snail life-cycle, to ensure relatively constant conditions concerning competition for space and food (see Online Resource, Fig. 2).

**Fig. 2:**
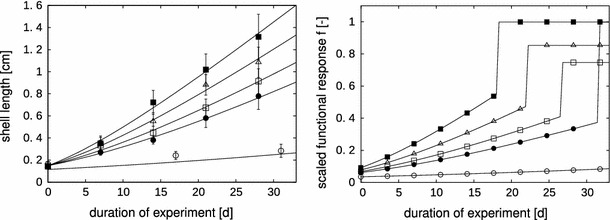
Growth of the juvenile pond snails in the full life-cycle experiment (FLE) and juvenile feeding experiment (JFE), and the corresponding model predictions (left panel). JFE: $\blacksquare$ maximum level fish flakes, ▵ medium level fish flakes, $\square$ minimum level fish flakes, $\bullet$ ad libitum lettuce; FLE: $\circ$ ad libitum lettuce. The scaled functional response *f* (as a proxy for food availability), resulting from the linear food limitation function (Eq. 3, Table 1) is presented as a function of time (*right panel*). The* symbols* on the model lines stand for the same regimes as in the* left panel*, but do not represent data points

The PLE was conducted to assess the impact of different food levels on the growth and reproduction of the pond snail. It was conducted under the same photoperiod and temperature as the FLE. In contrast to the FLE, this experiment started with juveniles (age 113 days and shell size 12.7 ± 1.3 mm) that had been reared under culture conditions. The size at the beginning of the PLE was chosen based on the outcome of the FLE: from a size of around 1cm, the snails in the FLE grew following the von Bertalanffy growth pattern. The snails were kept at three different constant food levels. The first regime was supposed to be ad libitum, where the amount of food given the first day was determined from the amount of lettuce that was eaten in the FLE from snails of similar size. After that, the amount of lettuce given was doubled each time when half of the regimes had consumed the previously given amount of lettuce. To determine the amount of lettuce that was consumed, the lettuce was weighted before feeding, and the leftovers on the next day were weighted as well. The second and third regime received half of that weight and a quarter of that weight.

### The juvenile feeding experiment (JFE)

To test the hypothesis of juvenile food limitation in the FLE, we conducted a juvenile feeding experiment (from now on JFE) with different types and quantities of food. We took clutches from the culture and let them develop under the same light regime as for the longterm experiments, but at a temperature of $23.5 \pm 0.75\,^{\circ} \hbox{C}$. We collected hatchlings of similar age (1 ± 1 day) and size (1.4 ± 0.1 mm) and transferred them into 100 ml test vessels in groups of five. The complete water volume was renewed weekly and they were fed three times a week with different amounts of lettuce or TetraPhyll$^{\circledR}$ fish flakes (see Online Resource, Table 4). We tested two regimes were the snails were fed ad libitum with lettuce. In one regime (Lettuce 1), sand was added, under the hypothesis that it would facilitate food digestion. The amount of lettuce given was determined in terms of numbers of slices of 21 mm Ø. The amount of fish flakes was chosen based on a recommendation for well-fed adults of *Potamopyrgus antipodarum* (OECD 2010), which reaches the same size as juveniles of *L. stagnalis* (≈0.5 cm). We used a value that was slightly higher than the recommended value (0.3 mg/day/ind., recommended 0.25) for the maximum food level as a starting value, and increased the amount given if all had been eaten on the next feeding day. The two lower levels were calculated as half and a quarter of the maximum amount. Food was only provided if no leftover remained. Shell size was measured weekly using a binocular microscope fitted with a micrometer. The experiment lasted 28 days.

### Obtaining model parameters and error structure from experimental data

All model parameters were estimated from the growth data of the FLE, PLE and JFE simultaneously, whereby the initial length *L*_0_ was estimated for each experiment. The food quality factor *a* (Eq. 3) was estimated for the different food sources: one *a* was estimated for fish flakes from the JFE, whereby a different *f*_0_ was estimated for each of the three food levels. In both the JFE and the FLE, lettuce was provided ad libitum, so that we set *f*_0_ = 1 in both experiments. However, we needed to estimate one *a* for the juveniles in the FLE (i.e., *L* < *L*_*f*_), and a different *a* for the lettuce-fed regime in the JFE to be able to capture the observed patterns. Note that we use the same *L*_*f*_ for lettuce and fish flakes since the JFE was too short to estimate a separate value the fish flakes experiment treatment.

The estimation routines were implemented in Matlab 2010a; scripts to perform these calculations can be found on http://www.debtox.info/debtoxm.php. We used maximum likelihood optimization, and derived the confidence intervals by profiling the likelihood (e.g., Meeker and Escobar 1995). For the maximum likelihood estimation, we need to make an assumption for the error structure of the data. An analysis of the scatter structure of the data showed that the variance increased with mean shell length for the PLE and the JFE (see Online Resource, Fig. 3). However, the error structure of the FLE shows a different pattern: the growth data has a high variance in the fast growth phase, and a lower variance in the slower growth phases, i.e. at the beginning and at the end of the experiment (see Online Resource, Fig. 3). We used an empirical spline function to describe the variance as a function of length, and used the actual error pattern in the likelihood function (see also Jager and Zimmer 2012).

**Fig. 3:**
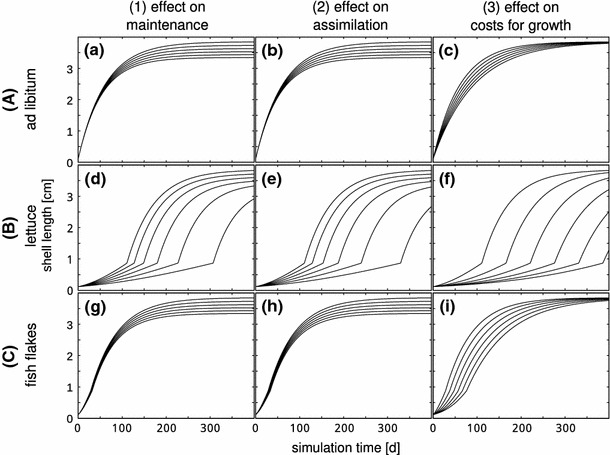
The simulation of growth curves of snails exposed to the hypothetical toxicants. From* left to right*, the different mechanisms of effect are shown, while from* top to bottom*, the feeding scenarios are displayed. Scenarios:** a**–**c** without food limitation;** d**–**f** with the linear food limiting function, assumed to be fed on lettuce;** g**–**i** with the linear food limiting function, assumed to be fed with fish flakes.* Top line*: control conditions.* Lines from top to bottom* represent the scenarios were the respective parameters are changed

## Results

### Experiments and model fit

The growth curves from the FLE and the PLE and the corresponding model fits are shown in Fig. 1.

The growth pattern in the FLE cannot be reproduced with the standard DEB model in constant environmental conditions which follows the von Bertalanffy growth pattern (Fig. 1, left panel). With the juvenile food limitation (see Eq. 3), the pattern can be captured. The PLE was started with larger individuals and the growth pattern follows the von Bertalanffy pattern from the beginning (Fig. 1, right panel). The corresponding model parameters are shown in Table 1. We use shell length as our measure of body size, which implies that all parameters with length in their dimension (including $\dot{v}$) are based on this size measure. Note that the maximum shell length reached by the snails fed with the maximum food level in the PLE is lower than the one in the FLE, although both were supposed to be fed ad libitum. Thus, the scaled functional response that was estimated for the PLE is smaller than one (see Table 1).

**Table 1:** Model parameters and values as fitted simultaneously using maximum likelihood optimization from the FLE, PLE, and JFE. Confidence intervals were derived using profile likelihoods

Parameter	Unit	Value	95 % conf. interval	Definition
$\dot{k}_M$	d^−1^	0.4882	0.3246–0.8781	Somatic maintenance rate coefficient (21 °C)
$\dot{v}$	cm d^−1^	0.2121	0.197–0.227	Energy conductance (21 °C)
*g*	–	0.1176	0.0627–0.1821	Energy investment ratio
*L*_*f*_	cm	0.8687	0.7718–0.9789	Critical length
*L*_01_	cm	0.1151	0.07589–0.165	Initial length FLE
*L*_02_	cm	1.212	1.154–1.271	Initial length PLE
*L*_03_	cm	0.1491	0.1385–0.1596	Initial length JFE
Food level FLE				
*f*	–	1	–	Ad libitum feeding with lettuce (fixed to one)
Food level PLE				
*f*_1_	–	0.8579	0.8236–0.9145	Highest level
*f*_2_	–	0.7505	0.7311–0.7736	Medium level
*f*_3_	–	0.6679	0.6431–0.6733	Lowest level
Food level JFE				
*f*_01_	–	1	–	Highest quantity fish flakes (fixed to one)
*f*_02_	–	0.8549	0.8247–0.8813	Middle quantity fish flakes
*f*_03_	–	0.7482	0.7166–0.7745	Lowest quantity fish flakes
Food quality factor				
*a*_*tet*_	–	2.359	2.198–2.577	Food quality factor fish flakes
*a*_*let*1_	–	1.235	1.183–1.303	Food quality factor lettuce, FLE
*a*_*let*2_	–	1.61	1.548–1.688	Food quality factor lettuce, JFE

The growth curves of the JFE are shown in Fig. 2 (left panel). The newborn pond snails grow much faster when fed with fish flakes, and reach double the size of the lettuce-fed snails after four weeks. Note that we used the mean growth of regime Lettuce 1 and 2, since they were not significantly different (see Online Resource, Table 5). Additionally, there is a difference between the juveniles fed with lettuce in the JFE compared to the juveniles in the FLE: the juveniles in the FLE only reached half the size of the juveniles in the JFE after four weeks, which is reflected in the difference between *a*_*let*1_ and *a*_*let*2_. The estimated food quality factors *a* as well as the constant scaled functional responses *f*_0_ for each regime are listed in Table 1. The scaled functional responses resulting from Eq. 3 over time are presented in Fig. 2 (right panel). Note that *f(L)* is still between 0 and 1 by definition.

### Model simulations

The simulated growth curves for the scenarios with the three hypothetical toxicants are shown in Fig. 3.

In each graph, the top line represents the simulation with the default parameter, and the lines from top to bottom represent simulations with the changed parameter value. Note that for effects on maintenance (1) and assimilation (2), the parameters were increased up to 15 %, so that each next line denotes an increase of the corresponding parameter value of 3 %. For the effect on costs for growth, the parameter was increased up to 600 %, so that each next line denotes an increase of 100 %. An effect on maintenance (left panel, (1)) decreases the maximum size (see Fig. 3a). While the initial growth is hardly influenced in the fish flakes scenario (see Fig. 3g), the initial growth of the lettuce fed juveniles is strongly impacted (see Fig. 3d), and the maximum size is only reached at the end of the simulation time for all levels of impact. The effect on assimilation (middle panel, (2)) strongly resembles the effect on maintenance: the maximum size is decreased under real ad libitum feeding conditions (see Fig. 3b). However, for the lettuce scenario (e), juvenile growth is stronger impacted than with an effect on maintenance with the same percentage of effect (d). The effect on costs for growth (right panel, (3)) shows a different pattern: the maximum size is not impacted, but the growth rate is decreased (see Fig. 3c). When fed with fish flakes, the effect is slightly stronger in the early juvenile phase (see Fig. 3i). However, when fed with lettuce (see Fig. 3f), the growth is strongly impacted in the juvenile phase, and the growth pattern resembles the two other toxicants, when fed with lettuce.

## Discussion

### Deviation of the von Bertalanffy growth pattern

The growth curve that was obtained in the FLE deviates from the von Bertalanffy pattern, and thus from the predictions of the standard DEB model in constant environmental conditions (Fig. 1). A deviation from the von Bertalanffy pattern could have two reasons: (1) the assumptions that underlie the model structure do not hold for the pond snail, or (2) the conditions, to which the model applies, are not met. The von Bertalanffy model applies to organisms that grow isomorphically (i.e., do not change in shape). Kooijman (1993) shows that the shell of the pond snail does grow without a change in shape. Thus, it should follow the von Bertalanffy growth pattern under constant conditions. The lack of this pattern in Fig. 1 (left panel) suggests that the snails do not experience the experimental conditions as constant. In nature, the pond snail can be considered a generalist, and its food consists of detritus and decomposed macrophytes (Kolodziejczyk and Martynuska 1980). In the adult stage, the main part of its food is thought to consist of macrophytes (Elger and Lemoine 2005), while juveniles and hatchlings probably mainly feed on periphyton. This switch in diet is not accounted for in the experimental setup. Therefore, we hypothesize that the snails do not experience a constant food level, although they are constantly fed with an ad libitum amount of lettuce. Instead, the juveniles are limited, either by the composition or accessibility of the provided food. The outcome of the JFE supports our hypothesis: the newborn pond snails grow much faster when fed with fish flakes, and reach double the size of the lettuce-fed snails after four weeks (Fig. 2).

### Food limitation in the pond snail

The simplest relation that could provide a good description of the growth pattern was a linear function of body size (see Eq. 3). With this function, we were able to capture the observed pattern with the DEB model (see Fig. 1).

With the linear food quality factor, we can qualitatively compare the nutritional status of the juveniles in the JFE: it is higher for fish flakes than for lettuce (see Table 1). These differences might be due to the accessibility of the provided food. Fish flakes have a very low density: they first float on the water and sink to the bottom of the test vessels when they soak up enough water. In contrast, the lettuce only floats on the water surface. The pond snail is a grazer, and searches for food on all surfaces of the test vessel (i.e., water surface and vessel walls). The fact that the fish flakes are both on the water surface and on the bottom of the vessel increases the chance of the juvenile snails to find a food item. Moreover, the small size of fish flakes might facilitate the uptake by the small mouth openings of the juvenile snails.

Apart from accessibility, the food limitation might as well result from the composition of the provided food. Among the types of food that have been tested in the laboratory, dry matter content (DMC) and protein content are the two main determinants of food preference for the pond snail. In a study on the palatability of macrophytes, the two species with the highest protein content in combination with the lowest DMC were the most appealing to the snails (*Berula erceta*: DMC 11.3 %, protein 10.8 %, and *Sagittaria sagittifolia*: DMC 5.4 % and protein 12.8 %, see Elger and Lemoine 2005). The composition of lettuce is in general similar to macrophytes, and seems to fulfill the requirements of the adult pond snails: the protein content of lettuce varies between 20 % (McKeehen et al. 1996) and 40 % (Selck et al. 2006), whereas the DMC varies between 5 and 12 %. In the juvenile stage, pond snails might be mainly feeding on periphyton and biofilms. For both, the protein content and DMC highly depends on the species composition and substrate. The protein content of biofilm can vary between 5–20 %, depending on the species composition, (Da Silva et al. 2008),while the protein content of periphyton can vary between 13–32 %, depending on the substrate (Azim et al. 2002). The DMC of periphyton can be around 10 % (Sladecek and Sladeckova 1963). The protein content of TetraPhyll $^{\circledR}$ fish flakes given by the manufacturer is 46 %. The high protein content and softness of the fish flakes seems to make it a better food for the juvenile snails than lettuce. Yet, further experiments are needed to investigate the effects on the rest of the life-cycle.

### Protein content of food in other aquatic invertebrates

Finding the right food type to culture aquatic organisms is a challenge. The influence of different food sources is mainly studied concerning the growth of animals, not only in eco(toxico)logical experiments (e.g., Ristola 1995; e.g., Egeler et al. 2010), but also in bioproduction for human consumption (e.g., Van Dam et al. 2002). In aquaculture, juvenile growth under laboratory conditions has been studied intensively, and recently higher growth efficiency has been linked to protein content of the food (e.g. for queen conch *Strombus gigas*, Garr et al. 2011). Additionally, in the snail *Potamopyrgus jenkinsi*, lamb heart versus lettuce has been tested (Dorgelo et al. 1995), and in *Marisa cornuarietis*, growth in the juvenile phase has been investigated using Tetramin $^{\circledR}$, baby cereals and spinach (Selck et al. 2006). In both studies, the motivation was to test types of food with higher protein content than lettuce, and to compare the performance. However, only in *M. cornuarietis*, fast growth could be directly linked to protein content, while in *P. jenkinski*, protein content was not the only determinant for fast growth: the snails grew fastest on a mixture of lambs heart and lettuce (Dorgelo et al. 1995).

### Differences in patterns following from experimental setup

Interestingly, the juveniles in the FLE grow much slower than the slowest ones in the JFE, although both are fed ad libitum with lettuce (see Fig. 2). The experiments have been conducted under different temperatures: the FLE at 21°C, and the JFE at 23.5°C. All rate constants in organisms tend to depend on temperature in a very similar way (Gillooly et al. 2006). By including the temperature dependence of the rate parameters $\dot{k}_M$ and $\dot{v}$, part of the difference in growth can be explained. However, even with the inclusion of the temperature dependence, we need a lower food quality factor in the FLE than in the JFE to be able to capture the growth pattern. One reason might be a difference in the quality of the provided lettuce: the nutrient content of lettuce is known to vary with growing season (Fallovo et al. 2009), and the two experiments were started at different times of the year (see Online Resource, Sect. 2.3).

Surprisingly, the snails in the FLE and the PLE reach a different maximum length, although both are supposedly fed ad libitum with lettuce. The reason for the difference might be a difference in the determination of the ad libitum feeding regime in the two setups. The amount of lettuce was doubled whenever half of the replicates had eaten all that was provided the day before in both setups. However, while in the FLE the amount given was determined in terms of surface area (i.e. number of slices), in the PLE the amount was determined by weight (see Online Resource, Sect. 2.3). Additionally, the maximum size of the snails in both experiments is much smaller than the maximum size of *L. stagnalis* as observed in nature (≈6 cm, see Online Resource 2.1). Both the FLE and the PLE may thus not have represented ad libitum feeding conditions for adults.

### Model simulations

The DEB formulation of the von Bertalanffy growth model allows us to study possible implications of the food limitation for ecotoxicological tests. The simulation studies in Fig. 3 show that food limitation has synergistic effects with all tested hypothetical toxicants. Under real ad libitum conditions, the effect of the toxicant is not very pronounced: for effects on maintenance costs and assimilation, the maximum size is reduced slightly (Fig. 3a, b), while with an effect on costs for growth, the growth rate is decreased, but there is no effect on ultimate length (Fig. 3c). In the simulations where the juveniles are assumed to be fed with lettuce, and thus food limited, the same degree of effect on the target parameter leads to stronger effects on growth. In addition to the effects on growth observed in the simulations, the juvenile phase is prolonged by all hypothetical toxicants (Fig. 3d, e, f). In organisms that start reproducing at a given size (e.g., the pond snail), a decrease in juvenile growth would imply a delay in the start of reproduction, which can have a substantial impact on the population growth rate (Kammenga et al. 1996). Moreover, even for organisms that start reproducing at a given age, the population would be affected: smaller organisms eat less and have less energy available to invest in reproduction than larger organisms. In the simulations where the juveniles are assumed to be fed with fish flakes, there is hardly any visible additional effect (Fig. 3g, h, i). Only for the simulation with an increase in the costs for growth, the juvenile phase is slightly prolonged.

The simulation studies thus indicate that juvenile pond snails feeding on lettuce may show larger response to the same toxic stress than juvenile snails feeding on fish flakes. If we are not aware of the food limitation, this can lead to an overestimation of the toxicity of the tested compound. However, the interaction between the food limitation and the chemical depends on its mMoA.

### Implications for ecotoxicology and risk assessment

Eventually, we want to assess the impact of a compound on the test organisms under natural conditions. Since organisms hardly ever experience unlimited food conditions in nature, the response under food limitation might be a more realistic representation of toxicity. However, in the test system we describe (the pond snail fed on lettuce), the degree of food limitation changes with the size of the snails. In mechanistic modelling of the toxic effect with DEB, we can account and compensate for that fact in the interpretation of the data (see Jager and Klok 2010). However, when using a descriptive analysis of the data (such as the ECx from a dose–response curve), the size-dependent (and thus also time-dependent) feeding limitation in a toxicity test will lead to bias in the results. The simulation study demonstrates that an unrecognised food limitation can lead to serious overestimation of toxicity, compared to the effects under good nutritional status. Even though food limitation may be realistic, such a bias in our toxicity data hampers the comparison of toxicity between chemicals and between species. The interaction between food limitation and toxicants is strong and ecologically relevant, but we need to study it in a controlled way. We need to separate the effects of the toxicant from the effects of the food limitation to be able to understand the mechanisms behind their interaction. Only then we can develop reliable models that can predict effects under different feeding scenarios, and thus support a scientifically sound risk assessment.

## Conclusions

In this study, we demonstrate the importance of food selection for ecotoxicological experiments. We showed that *L. stagnalis* is food limited in the juvenile phase, under conditions which are under consideration as standard conditions for OECD guidelines. Our simulation studies show that food limitation exaggerates the response to toxic stress. The interpretation of the results of life-cycle experiments (which include the early juvenile phase) may thus be biased when lettuce is used as the sole food source. Alternative food sources should be considered to avoid potential overestimation of toxicity. By using a mechanistic effect model (e.g. DEB), we can include the observed food limitation in the analysis and interpretation of test results. This modelling approach could thus potentially serve as a tool for extrapolating to other environmental situations for ecological risk assessment. Although food limitation is an ecologically relevant stress factor, we need to be able to separate these effects from the toxicity of the chemical stressor to make sense of the underlying mechanisms. Growth curves that deviate from the von Bertalanffy pattern are a good indication (but not a proof) of experimental problems with the food supply. Since a change in diet is a common strategy among organisms, this phenomenon does not only apply to the pond snail. So ecotoxicologists, be warned!

## Electronic supplementary material

Below is the link to the electronic supplementary material.

PDF (519 KB)
