# Supplementary material for: Juvenile food limitation in standardized tests: a warning to ecotoxicologists
Source: Ecotoxicology. 2012 Jul 28;21(8):2195–204. doi: 10.1007/s10646-012-0973-5 (PMC3475972; doi:10.1007/s10646-012-0973-5)
Supplement: Supplementary file 1 — PDF (519 KB) [file 10646_2012_973_MOESM1_ESM.pdf]

Electronic supplementary material to: Juvenile food limitation in standardized tests - a warning to ecotoxicologists. Published in *Ecotoxicology*

E.I. Zimmer*, T. Jager, V. Ducrot, L. Lagadic, S.A.L.M. Kooijman

Contents

1 Dynamic Energy Budget (DEB) theory **3**

1.1 Concepts and ideas 3
1.2 The model 3
1.3 Inclusion of toxic effects 8
1.4 Temperature dependence 9

2 Experimental part **10**

2.1 Life-cycle of *Lymnaea stagnalis* 10
2.2 Rearing conditions at the snail culture 10
2.3 Experiments 10
2.3.1 The full life-cycle experiment (FLE) 10
2.3.2 The partial life-cycle experiment (PLE) 13
2.3.3 The juvenile feeding experiment (JFE) 13

3 Analysis of the error structure **16**

List of Figures

S1 The standard DEB animal model 5
S2 Reserve dynamics follows food level 8

*Dept. Theoretical Biology, Fac. Earth & Life Sciences. Vrije Universiteit, de Boelelaan 1085, NL-1081 HV Amsterdam, The Netherlands. Email: Elke.Zimmer@vu.nl

S3 Scheme of the FLE setup 12
S4 Variance over shell length 16

List of Tables

S1 The DEB model parameters 6
S2 The three metabolic mechanisms of action (mMoA) 9
S3 Growth data FLE and PLE 14
S4 Feeding data JFE 15
S5 Growth data JFE 15

1 Dynamic Energy Budget (DEB) theory

1.1 Concepts and ideas

Dynamic Energy Budget (DEB) theory provides a set of rules that capture how much energy organisms assimilate from food, and how this energy is allocated to growth, development, reproduction and maintenance. It was originally developed with the aim to understand how organisms change the allocation of energy in response to a toxicant (Kooijman and Metz, 1984): when a daphnid eats the same amount of algae, but produces less offspring, the energy has to be invested for something else. Following the idea that the energy metabolism is organized very similarly among organisms, DEB can in general be applied to all organisms (Kooijman, 2001; Nisbet et al, 2000). The general DEB animal undergoes three life stages: embryo, juvenile and adult. During each stage, organisms basically follow the same rules for energy allocation, but the switch between life stages indicates a switch in metabolic behavior. The embryo is defined as an organism that does not feed and only lives from the reserves that were handed over from the mother. Once it starts feeding (first switch), it is considered a juvenile. When it reaches maturity and starts reproducing (second switch), it is considered an adult until it dies. However, for some organisms, additional life-stages have been included, and for others only two of the three stages are needed (Kooijman, 2010). Models for other organisms than animals, such as plants and bacteria, have been developed as well. Here, we only consider the standard animal model because it has been applied in the present work.

1.2 The model

In DEB, the biomass of an organism is composed of structure and reserve, which are two of the three state variables in the standard DEB model. Structure consists of all parts of the organism that require maintenance (e.g. cell membranes). Reserve captures all parts of the organism that fuel other metabolic processes, but do not require maintenance. The third state variable is the most abstract one: maturity. Maturation relates to an increase in complexity of the organism, and determines the switches between life stages in DEB. A certain amount of energy needs to be invested into maturation before the embryo can become a juvenile, i.e., start feeding, and before the juvenile can become an adult, i.e., start reproducing. All energy fluxes are fully determined by 12 primary parameters, which translate to measurable quantities via compound parameters (see e.g., Kooijman et al, 2008). Here, we are focusing on the energy fluxes from food into reserves and from re-

serves into growth and maintenance in juveniles. Therefore, maturity and reproduction do not play a role in the present study.
Structure is described in terms of structural body volume V (cm^3). The cubic root of the structural volume is the structural length L (cm). In the paper, we assume that shell length is proportional to structural length. Reserves are followed in terms of energy E (J). Before we dive into the details of the energy fluxes, we should specify a few more DEB parameters that we did not mention in the article, where we only mention g, the energy investment ratio $(-)$, $\dot{v}$, the energy conductance $(\mathrm{cm\,d^{-1}})$, $\dot{k}_M$, the somatic maintenance rate coefficient (d^{-1}), and the scaled functional response f $(-)$. The scaled functional response f is the actual ingestion rate of an animal divided by the the maximum ingestion rate for its size. For an individual under *ad libitum* feeding conditions, $f = 1$, whereas for a starving individual, $f = 0$, so that for limiting conditions $0 < f < 1$.
The parameters $\dot{k}_M$ and g are compound parameters in DEB, i.e. parameters that can be expressed in terms of primary parameters:

$$\dot{k}_M = \frac{[\dot{p}_M]}{[E_G]} \quad \text{and} \quad g = \frac{[E_G]\dot{v}}{\kappa\,\{\dot{p}_{Am}\}} \tag{1}$$

In DEB, the notation already indicates in what unit parameters are defined. For example, parameters that are defined per unit of structure are always written between square brackets (see Table S1). Here, some primary parameters appear, such as $[E_G]$, the costs for one unit of structure, $[\dot{p}_M]$, the maintenance that needs to be paid for one unit of structure, and $\{\dot{p}_{Am}\}$, the maximum surface area specific assimilation rate. The parameter $\{\dot{p}_{Am}\}$ is a species specific parameter that determines the maximum rate at which the energy that is extracted from food can be assimilated into reserves (more information about general DEB notation can be found under http://www.bio.vu.nl/thb/research/bib/Kooy2010_n.pdf). The parameter κ, $(-)$, determines the fraction of the mobilized energy that is allocated to the soma (i.e. somatic maintenance and new structure).
The energy fluxes in DEB are always noted as $\dot{p}_i$, where the i stands for the different fluxes. The fluxes are represented in the model scheme in Fig. S1. Let us follow the energy that is taken up from food, and see how much of it is converted into new structure. The energy that is assimilated from food $(\dot{p}_A)$ is defined as follows:

$$\dot{p}_A = fL^2\{\dot{p}_{Am}\}. \tag{2}$$

Assimilation is assumed to be proportional to a square of the structural length L, based on a simple physical principle: mass transport from one

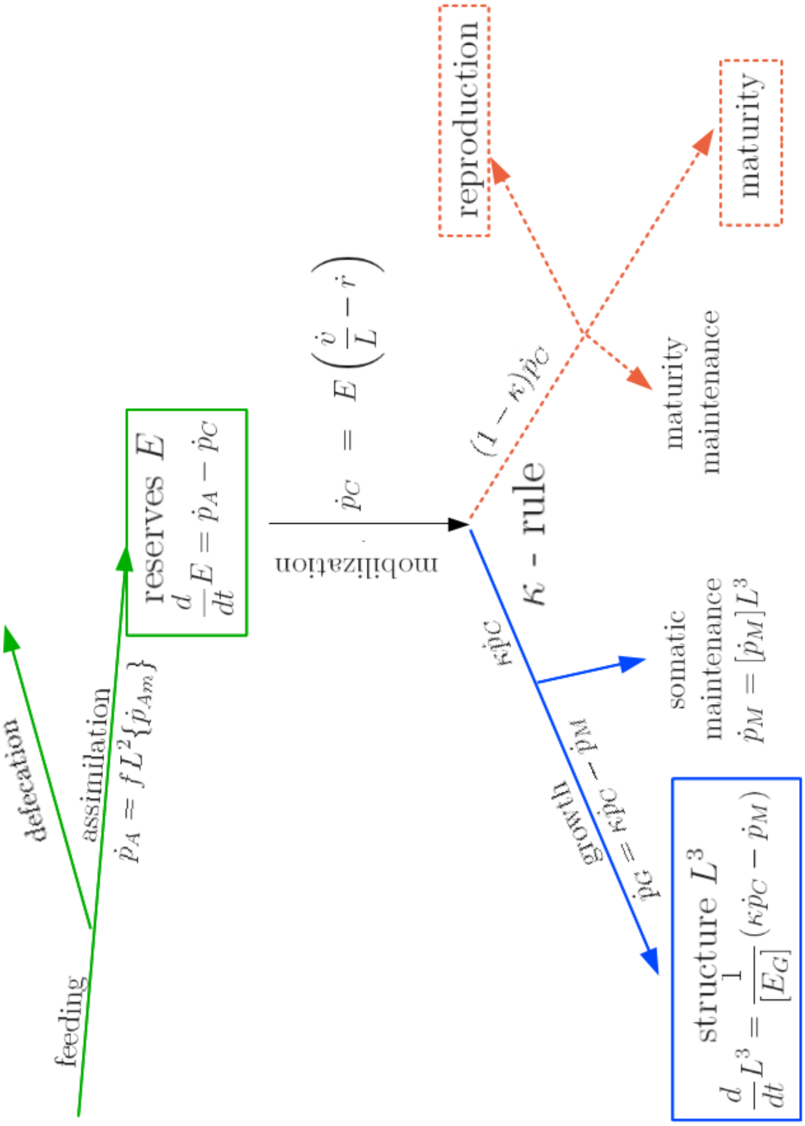

Figure S1: The standard DEB animal model, see text for a description.

Table S1: DEB model parameters used in this article with their symbols, interpretation and units.

Symbol	interpretation	units
**state variables**		
E	reserve	J
L	(stuctural) body length	cm
**primary parameters**		
$[E_G]$	volume-specific costs for structure	$\mathrm{J\,cm^{-3}}$
$[\dot{p}_M]$	volume-specific somatic maintenance	$\mathrm{J\,cm^{-3}\,d^{-1}}$
$\{\dot{p}_{Am}\}$	surface-specific maximum assimilation	$\mathrm{J\,cm^{-2}\,d^{-1}}$
$\dot{v}$	energy conductance	$\mathrm{cm\,d^{-1}}$
κ	fraction of mobilized reserves allocated to the soma	–
s	stress factor (0 in control)	–
**compound parameters**		
f	scaled functional response $(0 < f < 1)$	–
g	energy investment ratio	–
$\dot{k}_M$	somatic maintenance rate coefficient	$\mathrm{d^{-1}}$
**powers / energy fluxes**		
$\dot{p}_A$	assimilation power	$\mathrm{J\,d^{-1}}$
$\dot{p}_C$	mobilization power	$\mathrm{J\,d^{-1}}$
$\dot{p}_M$	somatic maintenance power	$\mathrm{J\,d^{-1}}$

environment to the another, as for example to an organism, must be across a surface (for illustration see Kooijman, 2010). The assimilated energy is first fixed into reserves before it is mobilized. The main reason for this assumption lies in the observation that organisms can survive in times of starvation, and even continue growing and reproducing for a while. The mobilization flux $\dot{p}_C$ is defined as:

$$\dot{p}_C = E\left(\frac{\dot{v}}{L} - \dot{r}\right) \quad \text{with} \quad \dot{r} = \frac{1}{L^3}\frac{d}{dt}L^3. \tag{3}$$

Mobilization is proportional to the amount of available reserve E. The possibilities for the reserve mobilization are severely restricted by the weak homeostasis assumption, which implies that the reserve density (i.e. reserve E per unit of structural volume) at constant food does not change, and thus cannot depend on body size. The relative growth rate $\dot{r}$ is included in the expression for reserve mobilization to counteract dilution by growth to allow for weak homeostasis. Kooijman (2010) argues that Eq. 3 is the only formulation that satisfies all requirements.

The dynamics of the reserves are given as:

$$\frac{d}{dt}E = \begin{cases} -\dot{p}_C & \text{for embryos} \\ \dot{p}_A - \dot{p}_C & \text{otherwise} \end{cases} \quad \text{with } E(0) = E_0 \tag{4}$$

A useful quantifier for reserve is the scaled reserve density e, which is the relative amount of reserve an organism has compared to its highest possible reserve density. For a well fed organism, $e = 1$.

$$\frac{de}{dt} = (f - e)\frac{\dot{v}}{L} \tag{5}$$

Under constant food conditions, $f = e$, and the Von Bertalanffy growth pattern is followed. In that case, we do not need to consider reserve dynamics explicitly. Since the energy conductance is rather high compared to the standard value ($\dot{v}_{std} = 0.02\,\mathrm{cm\,d^{-1}}$), we can consider the reserve dynamics to be fast. Indeed, when considering reserve dynamics with this particular parameter combination, e follows f rapidly (see Fig. S2).

A fixed fraction κ is of the mobilized energy is invested into the soma, and the rest $(1-\kappa)$ is invested into maturation or reproduction. The energy available for the soma is used for somatic maintenance $\dot{p}_M$, and the rest is used to build up new structure (growth). Somatic maintenance is proportional to the amount of structural volume:

$$\dot{p}_M = [\dot{p}_M]L^3 \tag{6}$$

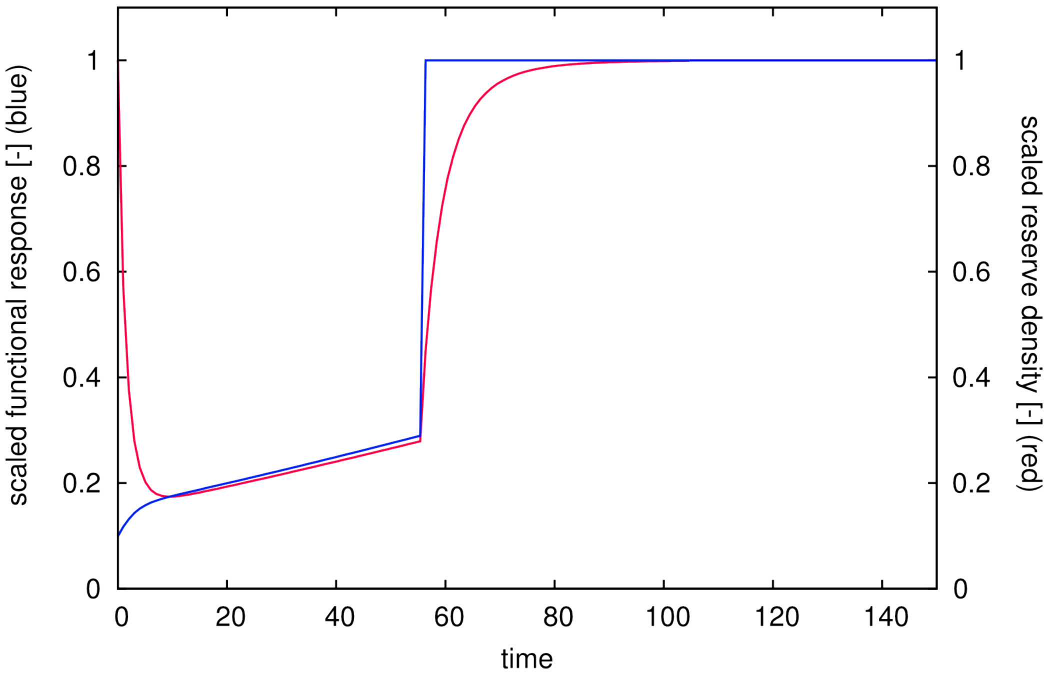

Figure S2: When reserves are included dynamically, the scaled reserve density e (red) follows the scaled functional response f (blue) very fast.

The energy available for growth is thus the energy available for the soma $\kappa \dot{p_C}$ minus the energy needed for somatic maintenance $\dot{p}_M$:

$$\dot{p}_G = \kappa \dot{p}_C - \dot{p}_M \tag{7}$$

The new structure that is build is the available energy divided by the costs for one unit of structure $[E_G]$:

$$\frac{d}{dt} L^3 = \frac{1}{[E_G]} (\kappa \dot{p}_C - \dot{p}_M) \quad \text{with } L(0) \approx 0 \tag{8}$$

To explain how we get from this equation to the Von Bertalanffy growth is beyond the scope of this paper. It can be found in Jager and Zimmer (2012). The energy that is not invested into the soma is used for maturation and reproduction (for more detail, see Kooijman et al, 2008).

1.3 Inclusion of toxic effects

In the article, we analyze three different metabolic mechanisms of action (mMoA). We are using compound parameters in the article, but the toxic effects are actually effects on primary parameters. Thus, we explain in the

Table S2: The three metabolic Mechanisms of Action (mMoAs) and their translation from effects on primary parameters into effects on compound parameters.

mMoA	primary parameter	compound parameter
(1) increase of maintenance costs	$\uparrow [\dot{p}_M]$	$\uparrow \dot{k}_M$
(2) decrease of assimilation	$\downarrow \{\dot{p}_{Am}\}$	$\downarrow f$
(3) increase of costs for growth	$\uparrow [E_G]$	$\downarrow \dot{k}_M, \uparrow g$

following how the effects of primary parameters translate to the compound parameters.

An effect on a parameter p can be expressed using the stress factor s . If the stress decreases the parameter, $p = p_0(1 - s)$, and if the stress increases the parameter, $p = p_0(1 + s)$, where p_0 is the parameter without stress (i.e., in the organisms in the control).
Using Eq. 1, we can now see how the above mentioned mMoAs are placed in the DEB model (see Table S2): effect (1) is actually an increase of the somatic maintenance costs per unit of structure $[\dot{p}_M]$, effect (2) is a decrease of the maximum surface area specific assimilation efficiency $\{\dot{p}_{Am}\}$, and effect (3) is an increase of the volume specific costs for structure $[E_G]$. From Eq. 2 we can see that f enters the assimilation flux in the exact same manner as $\{\dot{p}_{Am}\}$, so that an effect on both would result in the same effect pattern. Thus, it does not make a difference for the individual whether the effect is on $\{\dot{p}_{Am}\}$ or f. From ecotoxicological data, it is generally not possible to distinguish them.

1.4 Temperature dependence

In DEB, the temperature dependence of rate constants can be accounted for by multiplying them with a temperature correction factor c_T that can be derived from the Arrhenius relation (see Freitas et al, 2007). The temperature correction is given by

$$c_T = \exp\left(\frac{T_A}{T_{ref}} - \frac{T_A}{T}\right). \tag{9}$$

All temperatures are given in Kelvin. In Eq. 9, T_{ref} is the temperature for which we know the parameter value, T is the temperature for the new situation, and T_A is the Arrhenius temperature, which can be estimated if sufficient data is available. Since both the FLE and the PLE have been conducted at 21 ± 1°C, we set $T_{ref} = 273 + 21$. The JFE has been conducted

at $23.5 \pm 1°C$, so we set $T = 273 + 23.5$. For the Arrhenius temperature T_A, we use a typical value of $T_A = 11900$ (Kooijman, 2010). Typical data that would be suitable to estimate T_A would be respiration rates, ingestion rates or development times at different temperatures.

2 Experimental part

2.1 Life-cycle of *Lymnaea stagnalis*

Lymnaea stagnalis is a large freshwater snail with a maximum observed shell length of up to 6 cm (OECD, 2010) and a maximum observed age in the laboratory of 700 days (Slob and Janse, 1988). The hatching time highly depends on temperature, and ranges from 10 days (Horstmann, 1958) up to 27 days (pers. observation, FLE). It reaches maturity around 2-2.2 cm, with age at maturity depending on food conditions (Janse et al (1994), pers. observation).

2.2 Rearing conditions at the snail culture

The culture of *Lymnaea stagnalis* is kept at a constant water temperature of 20±1 °C and a constant light/dark period of 14/10 hours with a light intensity of 250-500 lux (natural daylight spectrum). We use dechlorinated charcoal filtered tap water, with the following properties: pH comprised between 7 and 8, oxygen concentration: > 6 mg/l at 20 °C, conductivity between 500 and 700 μS/cm. One third of the water volume is renewed weekly. Snails are fed three times a week with lettuce (organic quality, as certified by the european label ECOCERT, and washed in culture water). The amount of food delivered depends on the development stage, e.g. 160 g of fresh food/ 100 ind./week in adults. The maximum number of snails is 120 adults per 35 l.

2.3 Experiments

2.3.1 The full life-cycle experiment (FLE)

We use the growth data of a life-cycle experiment that has been conducted to investigate effects of diquat on the pond snail *Lymnaea stagnalis*. A detailed description of the experimental setup and part of the data has been published in Ducrot et al (2010).

The whole experiment was conducted under a photoperiod of 14/10 L/D using charcoal filtered tap water at $21 \pm 1°C$. Freshly laid clutches (less

than 24 h, laid on the 01/08/2007) were individually placed in plastic six-well plates (V = 10 ml). Hatching times were variable, but only newborns that hatched after 28 days were used for the life-cycle experiment. They were transferred to 100 ml Petri dishes (five snails per dish), where they were fed immediately. The snails were fed daily with weighted slices of organic lettuce, but only when no leftover from the day before remained, so that the risk of an effect on water quality due to disintegration of the lettuce was minimized. The amount of lettuce that was provided was gradually increased using the following rule: when the quantity given the day before was consumed in half of the replicates, the amount of lettuce given was doubled in terms of surface area. We started with one slice of 21 mm Ø in small juveniles. Then we doubled the Ø of the slices (from 21 to 43 mm). This resulted in the doubling of the leaf surface, and a corresponding increase by a factor of ca. 2.3 in weight. When lettuce was consumed in half of the replicates, we doubled the number of 43 mm slices that were provided and increased it to 2, then 4, and then 8 slices of 43 mm on day 168, which was the end of the contamination period in the regimes that were exposed to diquat. From that day on, the amount of lettuce given was not assessed anymore, but lettuce was provided *ad libitum*.

Water was regularly analyzed to determine physico-chemical characteristics regarding O_2, NH_4^+, NO_2^- and HPO_4^{2-} contents. Oxygen supply was not mandatory because the snail is a pulmonate. O_2 content in the water varied between 37.2% and 48.7% of the saturation value. In these conditions of water aeration, without any food leftover accumulating in the test vessels, weekly water renewals and the regular removal of feces with a pipette sufficed to maintain nitrogen products at values that are sufficiently low not to hurt the snails: e.g. NO_3^- varied in the range 7-15 mg/l and NH_4^+ varied in the range 0.02-0.5 mg/l.

The water volume was doubled each time when the amount of food was doubled. Water was renewed weekly during the whole experimental period. The volume of water that was renewed increased from 10% per week to 100% per week, depending on the amount of food that was consumed by the snails (the more they eat, the more feces they produce that needs to be removed). The actual volumes that were renewed as a function of age are indicated in Fig. S3.

To keep the number of snails per replicate constant, dead snails were removed daily from the vessels and replaced by siblings of similar age (±3 days) and size (±0.5 mm), which had been collected from the same clutches, and subsequently batch-reared under the same feeding conditions as the snails used in the life-cycle experiment. We kept batches of replacement snails for every tested concentration and controls. We stopped replacing snails when

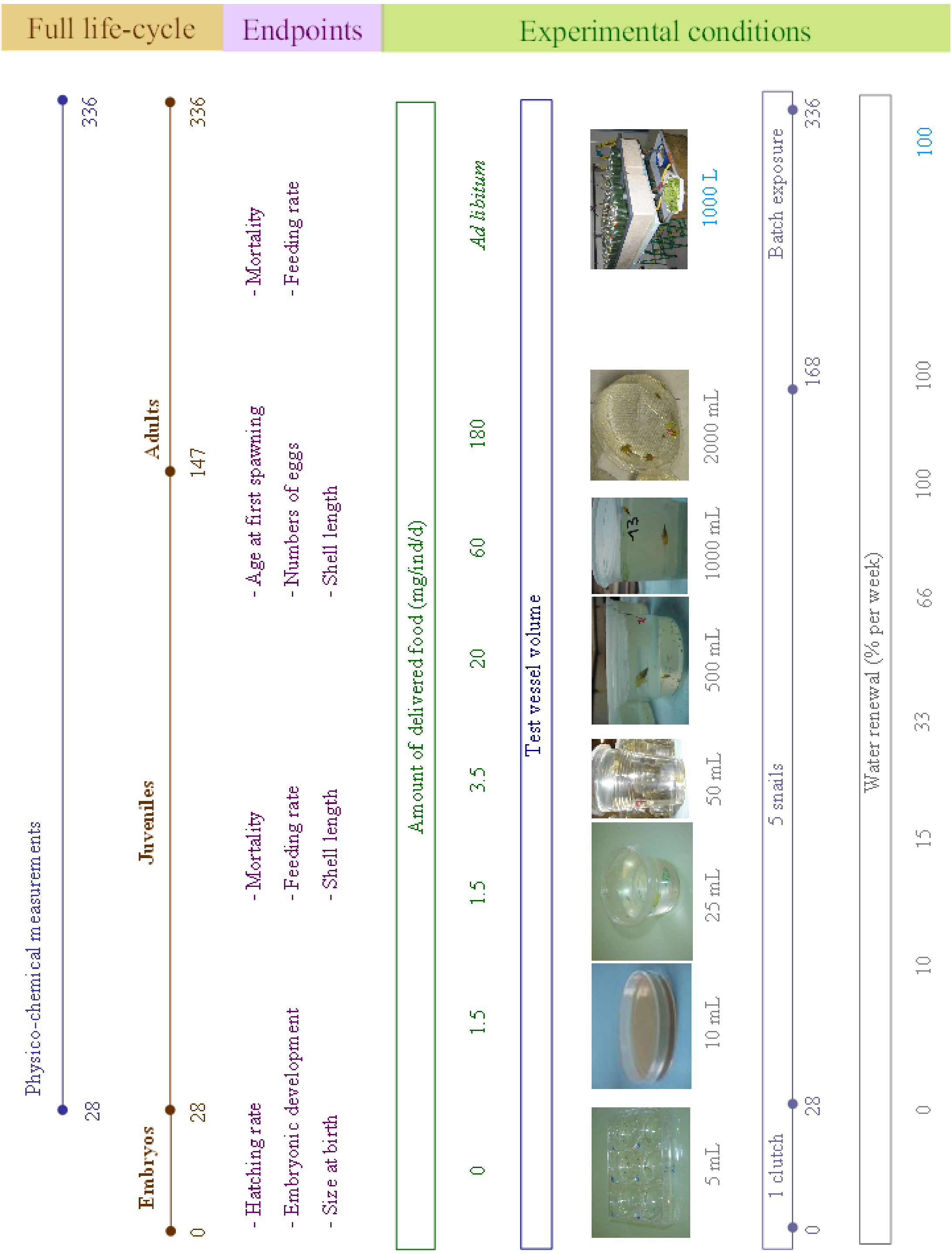

Figure S3: A schematic representation of the experimental setup of the FLE.

there were no replacement snails left that had the same size as the dead snail we wanted to replace (day 239). Shell length was measured every other week using a digital caliper (see Table S3). The experiment was stopped after 336 days, when survival fell below 75% (i.e., upper limit of the survival threshold for validity of toxicity test data, see American Society for Testing of Materials, 2000).

2.3.2 The partial life-cycle experiment (PLE)

The partial life-cycle experiment has been conducted to assess the effect of different food levels on growth and reproduction of the pond snail *L. stagnalis*. They were conducted under a similar protocol as was used for the FLE. The differences lie in the initial conditions, the duration of the experiment, the strategy of providing food, and the snail density per volume. The PLE was started with juveniles of homogeneous age (113 d) and similar size (12.7 ± 1.3 mm) that had been reared under culture conditions. The clutches were laid on the 15/09/2008, and the experiment started on the 06/01/2009. The juveniles were placed in 500 ml test vessels per groups of 5 (6 replicates per feeding regime). We tested four different food levels. The highest level was fed *ad libitum*, the second 50% of this quantity, the third 25 %, and the fourth was not fed. The initial value for the regimes with the highest food level was based on the results of the FLE: the snails received the same amount of lettuce as was given to the snails of a similar size in the FLE (80 g/ind. lettuce fresh weight). The amount of lettuce in this experiment was determined on a weight basis in contrast to slices (i.e. surface area) in the FLE. Each day, an *ad libitum* quantity of lettuce was weighted and given to the snails, and leftovers were weighted on the next day. The food for the regime with the second food level was determined as 50 % of the *ad libitum* value from the day before, and the third food level as 25 % respectively. In this experiment, dead snails were not replaced, so that the snail density per water volume slightly decreased over time. Shell length and wet weight were measured every other week, over 184 days. The complete water volume was renewed once a week.

2.3.3 The juvenile feeding experiment (JFE)

A detailed description of the juvenile feeding experiment can be found in the main text. The clutches were laid on the 22/06/2010. Details concerning the amount of food given in each feeding regime can be found in Table S4. The size at corresponding age is listed in Table S5.

Table S3: Mean shell size (mm), standard deviation (st. dev.) and numbers of individuals (# of ind.) of the FLE and the PLE. Note that day zero is defined as the day of egg-laying, and not as hatching.

	FLE			PLE *ad libitum*			PLE 50%			PLE 25%		
age	mean size [mm]	st. dev. [mm]	# of ind.	mean size [mm]	st. dev. [mm]	# of ind.	mean size [mm]	st. dev. [mm]	# of ind.	mean size [mm]	st. dev. [mm]	# of ind.
27	1.65	0.16	72	-	-	-	-	-	-	-	-	-
44	2.42	0.24	120	-	-	-	-	-	-	-	-	-
58	2.84	0.42	120	-	-	-	-	-	-	-	-	-
71	3.29	0.47	120	-	-	-	-	-	-	-	-	-
85	3.69	0.52	120	-	-	-	-	-	-	-	-	-
99	4.66	0.90	120	-	-	-	-	-	-	-	-	-
113	6.10	1.25	120	12.92	1.35	30	12.80	1.13	30	12.62	1.31	30
127	8.94	2.48	120	18.65	1.68	30	16.72	1.45	30	14.48	1.63	29
141	11.64	3.23	120	21.55	2.56	29	19.39	1.93	30	17.02	1.96	29
155	15.53	4.16	120	23.76	2.80	28	21.36	2.12	29	19.25	1.74	28
169	20.50	4.21	120	25.67	3.61	28	23.37	2.34	29	21.10	1.81	28
183	24.64	3.08	120	27.15	3.58	26	24.55	2.44	29	22.23	1.91	28
197	27.74	2.34	120	27.95	3.79	26	25.51	2.51	28	22.95	2.01	28
211	29.78	1.65	120	28.19	3.80	26	25.93	2.52	28	23.24	2.11	28
225	30.98	1.56	120	28.27	4.09	25	26.32	2.68	28	23.71	2.05	26
239	31.92	1.60	117	-	-	-	-	-	-	-	-	-
241	-	-	-	29.24	3.05	23	26.61	2.73	28	24.06	2.14	26
253	33.17	1.74	117	29.53	2.97	21	26.82	2.77	28	24.15	2.20	26
267	34.27	1.92	116	-	-	-	-	-	-	-	-	-
281	34.59	2.07	114	-	-	-	-	-	-	-	-	-
295	35.23	2.16	114	-	-	-	-	-	-	-	-	-
309	35.31	2.22	114	-	-	-	-	-	-	-	-	-
323	35.49	2.22	113	-	-	-	-	-	-	-	-	-
337	35.63	2.15	110	-	-	-	-	-	-	-	-	-

Table S4: The number of slices per replicate (i.e. 5 snails) and mg fish flakes per snail for the five experimental groups. Lettuce 1: without sand, Lettuce 2: with sand, Tetra 1: highest level of fish flakes, Tetra 2: mean level, Tetra 3: lowest level of fish flakes.

day	Lettuce 1 [# slices/rep.]	Lettuce 2 [# slices/rep.]	Tetra 1 [mg/ind.]	Tetra 2 [mg/ind.]	Tetra 3 [mg/ind.]
0	1	1	1.8	0.9	0.45
3	0	0	0	0	0.3
5	0	0	0	0.6	0.3
7	1	1	1.8	0.9	0.45
10	1	1	1.2	0.6	0.3
12	0	0	2.4	1.2	0.6
13	1	1	0	0	0
14	1	1	3.6	1.8	0.9
17	1	1	4.8	2.4	1.2
19	1	1	4.8	2.4	1.2
20	2	2	7.2	3.6	1.8
23	2	2	9.6	4.8	2.4
24	0	0	9.6	4.8	2.4
25	2	2	19.2	9.6	4.8

Table S5: Growth data and standard deviation of the juvenile feeding experiment (JFE). Lettuce 1: fed with lettuce without sand, Lettuce 2: fed with lettuce with sand, Tetra max: highest level of fish flakes, Tetra mid: middle level of fish flakes, Tetra min: lowest level of fish flakes.

	Lettuce 1		Lettuce 2		Tetra max		Tetra mid		Tetra min	
age	shell length	st. dev.	shell length	st. dev.	shell length	st. dev.	shell length	st. dev.	shell length	st. dev.
17	1.42	0.09	1.42	0.10	1.43	0.09	1.46	0.10	1.44	0.10
24	2.71	0.38	2.70	0.27	3.52	0.66	3.43	0.64	3.35	0.46
31	3.81	0.61	3.81	0.35	7.23	1.09	5.51	0.78	4.48	0.59
38	5.80	0.68	5.77	0.86	10.19	1.42	8.81	0.93	6.74	0.79
45	7.79	0.88	7.62	1.20	13.16	2.04	10.85	1.37	9.14	1.13

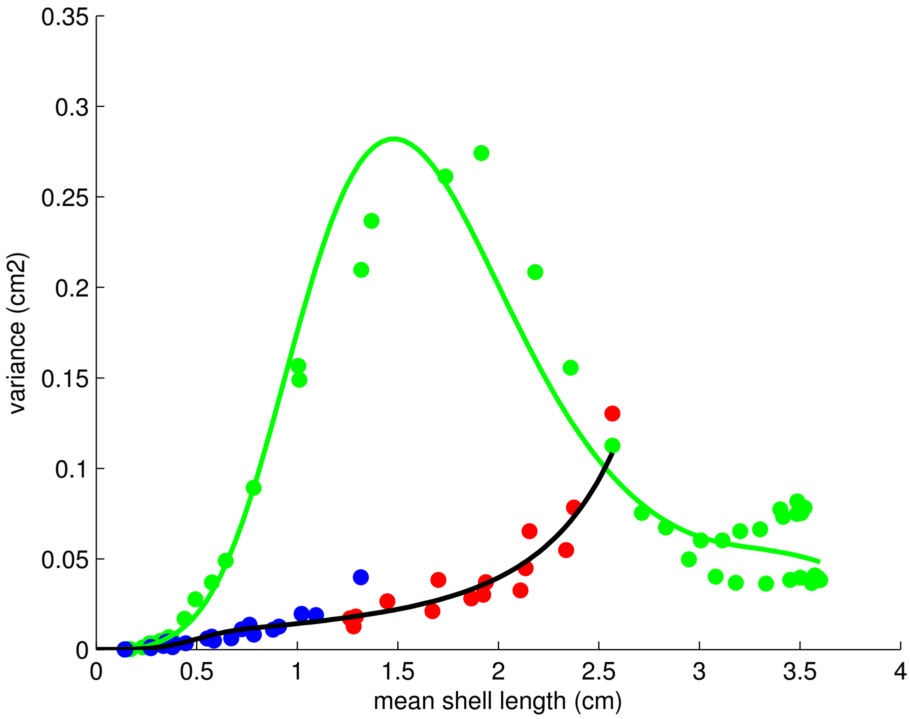

Figure S4: The mean variance over shell length of all experiments. Green: FLE, red: the PLE, blue: the JFE.

3 Analysis of the error structure

The error structure was different in the three experiments (see Fig. S4), so the error structure of the model used to describe the data was adapted accordingly. The variance increased with shell length in both the PLE and the JFE (i.e. large variance for large shell lengths). Interestingly, the variance of the FLE does not show this pattern: it is small for small shell length and small for large shell length, and has its peak around a shell length of 1.5−2 cm (see Fig. S4). One possible reason for this variation might be a difference in the way individual snails are limited by food: since the individuals are competing for food, a small difference in food generates a subsequent large difference in growth during the fast growth phase.

References

American Society for Testing of Materials (2000) Test method for measuring the toxicity of sediment-associated contaminants with freshwater invertebrates. ASTM Standards on Biological Effects and Environmental Fate 2nd Edition, ASTM (ed), Philadelphia, 18-23

Ducrot V, Péry ARR, Lagadic L (2010) Modelling effects of diquat under realistic exposure patterns in genetically differentiated populations of the gastropod *Lymnaea stagnalis*. Philos Trans R Soc B 365(1557):3485–3494, DOI 10.1098/rstb.2010.0047

Freitas V, Campos J, Fonds M, Van der Veer HW (2007) Potential impact of temperature change on epibenthic predator-bivalve prey interactions in temperate estuaries. J Therm Biol 32(6):328–340, DOI 10.1016/j.jtherbio.2007.04.004

Horstmann H (1958) Sauerstoffverbrauch und Trockengewicht der Embryonen von *Lymnaea Stagnalis* - L. Z Vgl Physiol 41(4):390–404[in german]

Jager T, Zimmer EI (2012) Simplified dynamic energy budget model for analysing ecotoxicity data. Ecol Model 225:74 – 81, DOI 10.1016/j.ecolmodel.2011.11.012

Janse C, Van Minnen J, Van der Roest M, Roubos E (1994) Female reproductive aging in the pond snail *Lymnaea-stagnalis*. Neth J Zool 44(3-4):385–394

Kooijman SALM (2001) Quantitative aspects of metabolic organization: a discussion of concepts. Philos Trans R Soc B 356(1407):331–349

Kooijman SALM (2010) Dynamic Energy Budget Theory for Metabolic Organisation. Cambridge University Press, Cambridge

Kooijman SALM, Metz JA (1984) On the dynamics of chemically stressed populations - the deduction of population consequences from effects on individuals. Ecotoxicol Environ Saf 8(3):254–274, DOI 10.1016/0147-6513(84)90029-0

Kooijman SALM, Sousa T, Pecquerie L, Van der Meer J, Jager T (2008) From food-dependent statistics to metabolic parameters, a practical guide to the use of dynamic energy budget theory. Biol Rev 83(4):533–552, DOI 10.1111/j.1469-185X.2008.00053.x

Nisbet R, Muller E, Lika K, Kooijman S (2000) From molecules to ecosystems through dynamic energy budget models. J Anim Ecol 69:913 – 926

OECD (2010) OECD Series of Testing and Assessment No. 121. Detailed review paper (DRP) on molluscs life-cycle toxicity testing. Organisation for Economic Co-operation and Development, Paris, France

Slob W, Janse C (1988) A quantitative method to evaluate the quality of interrupted animal cultures in aging studies. Mech Ageing Dev 42(3):275–290
